# Protective effect of budesonide/formoterol compared with formoterol, salbutamol and placebo on repeated provocations with inhaled AMP in patients with asthma: a randomised, double-blind, cross-over study

**DOI:** 10.1186/1465-9921-11-66

**Published:** 2010-05-28

**Authors:** René Aalbers, Martin Boorsma, Hanneke J van der Woude, René E Jonkers

**Affiliations:** 1 Department of Pulmonary Diseases, Martini Hospital, Groningen, The Netherlands; 2 Medical Department, AstraZeneca, Zoetermeer, The Netherlands; 3 Department of Pulmonary Diseases, Academic Medical Center, Amsterdam, The Netherlands

## Abstract

**Background:**

The budesonide/formoterol combination is successfully used for fast relief of asthma symptoms in addition to its use as maintenance therapy. The temporarily increased corticosteroid dose during increasing inhaler use for symptom relief is likely to suppress any temporary increase in airway inflammation and may mitigate or prevent asthma exacerbations. The relative contribution of the budesonide and formoterol components to the improved asthma control is unclear.

**Methods:**

The acute protective effect of inhaled budesonide was tested in a model of temporarily increased airway inflammation with repeated indirect airway challenges, mimicking an acute asthma exacerbation. A randomised, double-blind, cross-over study design was used. Asthmatic patients (n = 17, mean FEV_1 _95% of predicted) who previously demonstrated a ≥30% fall in forced expiratory volume in 1 second (FEV_1_) after inhaling adenosine 5'-monophosphate (AMP), were challenged on four consecutive test days, with the same dose of AMP (at 09:00, 12:00 and 16:00 hours). Within 1 minute of the maximal AMP-induced bronchoconstriction at 09:00 hours, the patients inhaled one dose of either budesonide/formoterol (160/4.5 μg), formoterol (4.5 μg), salbutamol (2 × 100 μg) or placebo. The protective effects of the randomised treatments were assessed by serial lung function measurements over the test day.

**Results:**

In the AMP provocations at 3 and 7 hours after inhalation, the budesonide/formoterol combination provided a greater protective effect against AMP-induced bronchoconstriction compared with formoterol alone, salbutamol and placebo. In addition all three active treatments significantly increased FEV_1 _within 3 minutes of administration, at a time when inhaled AMP had induced the 30% fall in FEV_1_.

**Conclusions:**

A single dose of budesonide/formoterol provided a greater protective effect against inhaled AMP-induced bronchoconstriction than formoterol alone, both at 3 and at 7 hours after inhalation. The acute protection against subsequent bronchoconstrictor stimuli such as inhaled AMP and the rapid reversal of airway obstruction supports the use of budesonide/formoterol for both relief and prevention in the treatment of asthma.

**Trial Registration:**

ClinicalTrials.gov number NCT00272753

## Background

The short-acting β_2_-agonist salbutamol is widely used as first-line treatment in the management of acute bronchoconstriction in asthma because of its fast onset of action [1]. The long-acting β_2_-agonist formoterol has an onset of effect that is comparable with that of salbutamol [2] and, when used as reliever therapy, has proven to be superior to terbutaline and salbutamol in improving asthma control and preventing asthma exacerbations [3-5]. The combination of budesonide and formoterol in one inhaler, used as maintenance treatment, improved asthma control compared with a similar or higher dose of an inhaled corticosteroid (ICS) alone [6,7]. Furthermore, budesonide/formoterol is also effective in situations of acute and severe bronchoconstriction [8,9], indicating that it is effective as a reliever therapy. Clinical studies have substantiated that budesonide/formoterol can be used as both maintenance and reliever therapy, resulting in improved asthma control and an additional reduction in exacerbation frequency compared with maintenance therapy plus a separate bronchodilator for relief [10-14]. The effectiveness of this novel treatment regimen, where patients use budesonide/formoterol as their only medication, is thought to be the result of a rapid increase in ICS dose at the earliest onset of symptoms [15].

A single dose of an ICS is thought to have limited bronchodilating effects and some immediate bronchoprotective effect [16,17]. In addition, an ICS has a vasoconstrictor effect in the airway mucosa, which can be measured within hours of administration [18].

Inhaled adenosine 5'-monophosphate (AMP) induces rapid degranulation of airway mast cells leading to bronchoconstriction and airway oedema and is, therefore, considered to mimic acute asthma attacks caused by allergen, cold air or exercise [19,20]. Bronchodilators can reverse AMP-induced bronchoconstriction and can also immediately protect against AMP-induced bronchoconstriction [21-23]. Long-term ICS treatment has a protective effect on bronchial hyperresponsiveness, as measured with inhaled AMP [24], but an ICS has also a small immediate protective effect against AMP induced bronchoconstriction, which lasts for several hours [25,26].

In daily life, patients with asthma can be repeatedly exposed to allergic and non-specific triggers resulting in airway constriction and asthma attacks. The present study was, therefore, designed to assess the protective effect of a single low dose of budesonide/formoterol with that of β_2_-agonist treatment only (formoterol or salbutamol) and placebo against repeated exposure to an indirect stimulus, AMP.

## Materials and methods

### Patients

Outpatients were included if they were: aged between 18 and 55 years with a diagnosis of asthma [1], had an FEV_1 _of >60% of predicted (26), used an inhaled corticosteroid in a dose of ≥ 100 μg daily, a provocative concentration of AMP causing a 20% fall in FEV_1 _(PC_20_-AMP) ≤160 mg/ml, and a demonstrated fall in FEV_1 _of >30% upon continuation of the AMP provocation. Patients had to be able to use and inhale correctly through Turbuhaler^® ^and a pressurised metered-dose inhaler (pMDI) connected to a large volume spacer device (Volumatic^®^); inhalation technique was practised until correct.

Patients were excluded from the study if, within 6 weeks prior to enrolment, they had used systemic corticosteroids, had experienced an asthma exacerbation or changed their ICS dose. Female patients who were pregnant, planning pregnancy, breastfeeding or not using an adequate method of contraception were also excluded. Patients were asked to avoid strenuous exercise, smoking and consumption of caffeine-containing beverages in the morning prior to the test days and throughout each of the test days. The study was performed in accordance with the ethical principles that have their origin in the Declaration of Helsinki and in accordance with Good Clinical Practice guidelines. The study was approved by the Medical Ethics Committees of both hospitals (Medical Ethics Committee Martini Ziekenhuis, reference number 2003-44 and Medical Ethics Committee Academic Medical Centre Amsterdam, reference number MEC 05/074. Written informed consent was obtained from all patients prior to their enrolment.

### Study design

**Figure 1:**
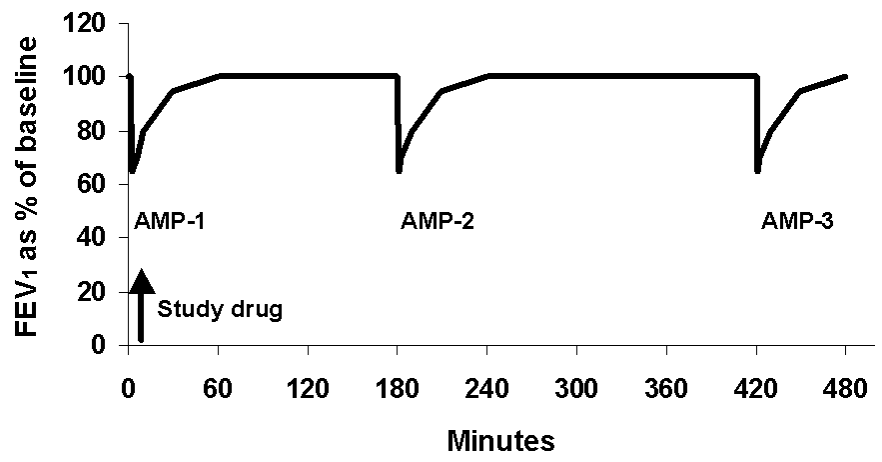
**Study design of the Test Days**.

This randomised, double-blind, double-dummy, placebo-controlled, cross-over study (study code BN-00S-0022, NIH ClinicalTrials.gov trial data base number NCT00272753) was conducted at two centres. The study comprised an initial enrolment visit at the start of the run-in period, a short visit at the end of the run-in period and four test days that were all separated by 5-14 days. The assessments on each test day are graphically shown in Figure 1.

At enrolment patients underwent an AMP provocation test with doubling concentrations of AMP increasing from 0.04 mg/ml to 160 mg/ml, inhaled during 2 minutes tidal breathing at intervals of 5 minutes until a fall from baseline FEV_1 _of ≥30% was demonstrated. Hereafter, ICS and bronchodilator treatment was standardised for the remainder of the study as once daily (in the evening) two inhalations of budesonide/formoterol 160/4.5 μg per dose (160/4.5 μg represents the delivered dose, this is 200/6 μg per metered dose, Symbicort^® ^Turbuhaler^®^, AstraZeneca, Sweden) and with terbutaline 250 μg per dose (Bricanyl^® ^Turbuhaler^®^, AstraZeneca, Sweden) for "as needed" use.

At the second visit, and after omitting the previous evening dose of budesonide/formoterol and abstaining from terbutaline use for 6 hours, responsiveness to inhaled AMP was confirmed in an abbreviated AMP provocation test, giving only the last four AMP doses that were given at enrolment (this was interrupted if FEV_1 _decreased ≥30%). Thereafter, on each of the subsequent four test days, three abbreviated AMP challenges were performed, commencing at approximately 09:00, at 12:00 and at 16:00 hours.

### Test-day assessments

The AMP provocation tests were only performed when the baseline FEV_1 _at 09:00 hours differed <15% from the value at enrolment and when FEV_1 _prior to each test was >60% of predicted. At the moment of completing the first AMP challenge on the test days, thus when there was an approximate 30% fall in FEV_1 _and within one minute of inhaling the last AMP dose, one of the double-blind treatments was inhaled: one inhalation of budesonide/formoterol 160/4.5 μg (via Turbuhaler^®^), one inhalation of formoterol 4.5 μg (via Turbuhaler^®^), two inhalations of salbutamol 100 μg (via pMDI connected to Volumatic), or placebo. On all occasions one inhalation from Turbuhaler^® ^and 2 inhalations from the pMDI were inhaled. Patients were randomised so that half of them used Turbuhaler^® ^for their first inhalation on each of the four test days and half used the pMDI first. Inhalers containing placebo or active medication had an identical appearance. The primary (FEV_1_) and secondary parameters (mean forced expiratory flow between 25% and 75% of forced vital capacity [FEF_25-75_] and the modified Borg scale [range 0-10] for perceived breathlessness [27]) were measured during the provocation test and at 1, 3, 5, 10, 15, 30, 45 and 60 minutes after each AMP provocation as well as hourly in between AMP provocations.

The highest values of three attempts of FEV_1 _and FEF_25-75 _were recorded [28] apart from during the first 20 minutes following AMP provocation when single assessments were made. During the abbreviated provocation itself, the lowest FEV_1 _(for safety reasons not the highest value was used) and the highest FEF_25-75 _of single assessments at 30 and 90 seconds after each 2-minute AMP inhalation were recorded.

### Statistical analysis

The primary aim of the study was to compare the magnitude of the bronchoprotective effects of budesonide/formoterol in comparison with formoterol alone. This was assessed as: (**1) **the maximal % fall in FEV_1 _in the 16:00 hours AMP provocation; (**2) **the mean % fall in FEV_1 _(calculated from the Area Under the FEV_1 _Curve (AUC_0-60_) from 0 to 60 minutes after the 16:00 hours AMP provocation); (**3) **the maximal % fall in FEV_1 _in the 12:00 hours AMP provocation; and (**4) **the Area Under the Curve on the entire Test Day, from 09:00 to 17:00 hours (AUC_9-17_) for FEV_1_. For the secondary parameters FEF_25-75 _and Borg Score only the AUC_9-17 _was calculated and compared. The % fall in FEV_1 _in the 12:00 and 16:00 hours provocation was expressed as % change from the baseline FEV_1_, measured immediately prior to that provocation to compensate for remaining bronchodilation from the study drug or remaining bronchoconstriction from AMP. For AUC_9-17_, the FEV_1 _and FEF_25-75 _values were expressed as % change from the test-day baseline value at 09:00 hours, the Borg score was expressed as absolute changes from the test-day baseline.

The onset of relief of bronchoconstriction by budesonide/formoterol after the first AMP provocation at 09:00 hours was expressed as the increase from the lowest FEV_1 _after AMP to the FEV_1 _at 3 minutes with both expressed as a % of baseline FEV_1_.

PC_20_-AMP values were calculated by interpolation from a log cumulative concentration versus % decrease in FEV_1 _response curve.

The AMP-induced change in FEV_1 _in the AMP provocation (as the ratio lowest/baseline FEV_1_) was compared between treatments in an additive analysis of variance model with subject, period and treatment as fixed factors and the test-day baseline FEV_1 _as covariate. Mean changes in FEV_1 _and two-sided 95% confidence intervals were calculated. Mean treatment differences were estimated by least-squares means resulting from this model. Other parameters were also analysed in this way. Of the above mentioned four ways to estimate the bronchoprotective effect, one parameter was chosen as the primary parameter in the power calculation prior to the study and in the statistical analysis: maximal % fall in the 16:00 hours AMP provocation. For this parameter, all six comparisons between the four treatments were tested. For all other parameters, statistical comparisons were restricted to the comparisons of budesonide/formoterol versus the three other treatments.

This study design with three AMP provocations on one each test day had not been used before. Therefore, sample size calculation was performed using data from a repeated cold air and exercise challenge study [29]. With an assumed standard deviation of 6.8% for the fall in the third AMP provocation and a power of 80%, a difference in the % fall in FEV_1 _of 4.5% would be detectable with 20 patients.

## Results

### Patients

Eighteen patients were randomised. One patient was withdrawn on the first test day prior to study treatment because of a baseline FEV_1 _below 85% of the FEV_1 _at enrolment, leaving 17 patients who received at least one dose of the study treatments. As a result of expiry of study drugs, no additional patients could be enrolled and two patients had to be withdrawn after completing two or three test days, respectively. Three test days were postponed because of unstable baseline lung function or use of non-allowed medication. No test day had to be interrupted for administration of bronchodilators. A summary of demographic and clinical data at enrolment is presented in Table 1. Baseline FEV_1_, the actual doses of AMP given and the resulting decrease in FEV_1 _and increase in Borg dyspnoea score prior to study treatment inhalation were very similar on each of the four test days (Table 2).

**Table 1:** Patient baseline demographics

Characteristic	Value
Sex: Male/Female, n	6/11
Mean age, years (range)	37.2 (20-53)
Median time since asthma diagnosis, years (range)	20.2 (3-42)
Mean inhaled corticosteroid dose prior to the study, μg (range)	553 (200-800)
User of long-acting β_2_-agonist prior to the study	14
Mean FEV_1_, L (range)	3.26 (2.11-4.69)
Mean FEV_1_, % predicted (range)	94.6 (63-126)
Geometric mean PC_20_-AMP, mg/ml (range)	2.64 (0.08-125)

FEV_1_: forced expiratory volume in 1 second; PC_20_: provocative concentration of adenosine 5'-monophoshate (AMP) causing a 20% fall in FEV_1_.

**Table 2:** Adenosine 5'-monophosphate provocation test data at 09:00 hours, immediately before administration of study treatments

Treatment	Baseline FEV_1_, before provocation (L)	AMP dose (mg/ml)	Fall in FEV_1 _after AMP provocation (%)	Increase in Borg dyspnoea score after provocation
Budesonide/formoterol	3.13 (0.80)	109 (184)	28.0 (14.0)	2.50 (1.40)
Formoterol	3.11 (0.86)	122 (193)	28.7 (12.2)	2.00 (1.35)
Salbutamol	3.03 (0.91)	110 (191)	31.6 (14.5)	2.91 (1.58)
Placebo	3.06 (0.89)	106 (186)	29.4 (10.6)	2.79 (1.57)

All data are presented as mean (SD); FEV_1_: forced expiratory volume in 1 second; AMP: adenosine 5'-monophoshate; AMP dose as cumulative nebulized concentration.

### Bronchoprotective effects

For the primary endpoint, the mean maximal fall after the third AMP provocation performed at 16:00 hours (i.e. 7 hours after treatment), was 15.7% after budesonide/formoterol, numerically (but not significantly) less than the 20.1% fall after formoterol (p = 0.24) and significantly less than the 29.8% and 31.9% fall after salbutamol (p = 0.0005) and placebo (p < 0.0001), respectively (Table 3). Formoterol alone provided significantly more protection (smaller fall in FEV_1_) than salbutamol (p = 0.014) and placebo (p = 0.0025) but salbutamol did not do better than placebo at 7 hours (p = 0.57).

**Table 3:** Protective effects of study treatments in repeated AMP provocations

	Fall in FEV_1 _in AMP provocation at 3 hours (%)	Fall in FEV_1 _in AMP provocation at 7 hours (%)	AUC_0-60_-FEV_1 _in AMP provocation at 7 hours (h.%)
Budesonide/formoterol	8.8 (4.0, 13.6)	15.7 (10.7, 20.8)	-4.2 (- 8.6, 0.2)
Formoterol	17.0 (11.8, 22.1)*	20.1 (14.6, 25.5)	-10.7 (- 15.4, -6.0)*
Salbutamol	20.1 (15.0, 25.2)^#^	29.8 (24.4, 35.2)^$^	-17.9 (- 22.5, -13.2)^$^
Placebo	27.1 (22.3, 31.8)^$^	31.9 (26.9, 36.9)^$^	-19.9 (- 24.3, -15.6)^$^

Data shown as Least Square Mean and 95% Confidence Interval; FEV_1_: forced expiratory volume in 1 second; AMP: adenosine 5'-monophoshate; fall in FEV_1 _as % from baseline prior to each AMP provocation; AUC_0-60_: Area Under the Curve for % change in FEV_1 _from 0 to 60 minutes after AMP provocation; p-values from ANOVA, differences compared with budesonide/formoterol: *p < 0.05, ^#^p < 0.01, ^$^p < 0.001.

The mean fall in FEV_1 _in the 60 minutes after the 16:00 hours AMP challenge (AUC_0-60_) was significantly smaller following budesonide/formoterol pre-treatment than that after formoterol (p = 0.045), salbutamol (p = 0.0001) and placebo (p < 0.0001) (Table 3).

All active treatments attenuated the bronchoconstriction by the AMP challenge at 3 hours after inhalation (i.e. 12:00 hours). The maximal % fall in FEV_1 _following budesonide/formoterol (8.8%) was significantly lower than that after formoterol (17.0%, p = 0.023), salbutamol (20.1%, p = 0.0028) and placebo (27.1%, p < 0.0001) (Table 3).

### Profile of FEV_1_, FEF_25-75 _and Borg score over the day

**Figure 2:**
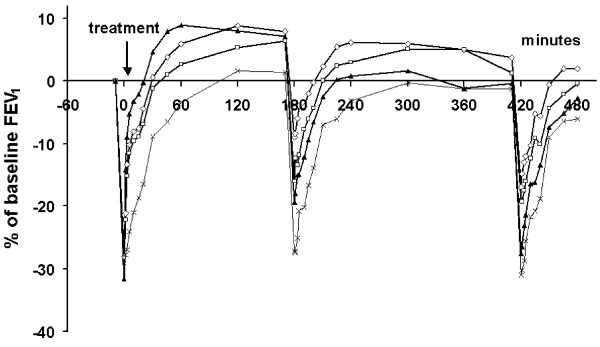
**Mean FEV_1 _over the test day with three AMP provocations followed by a single dose of budesonide/formoterol 160/4.5 μg (open diamonds), formoterol 4.5 μg (open squares), salbutamol 2 × 100 μg (filled triangles) or placebo (crosses) immediately after the first AMP provocation**.

The time course of FEV_1 _over the entire test day is presented in Figure 2. Initially, FEV_1 _was highest following 2 inhalations of salbutamol, but from 2 hours after inhalation onwards, FEV_1 _was highest following budesonide/formoterol. When calculated over the entire test day (as FEV_1 _AUC_9-17_), the FEV_1 _after budesonide/formoterol was significantly greater than that after formoterol (p = 0.033), salbutamol (p = 0.0011) and placebo (p < 0.0001, Table 4).

**Table 4:** Protective effects of study treatments in repeated AMP provocations over the entire Test Day

	AUC_9-17_-FEV_1_ (h.%)	AUC_9-17_-FEF_25-75_ (h.%)	AUC_9-17 _- Borg (h.units)
Budesonide/formoterol	20.9 (3.7, 38.1)	134 (69.8, 198)	0.21 (-1.64, 2.07)
Formoterol	-6.4 (-25.0, 12.1)*	47.8 (-21.5, 117)	-0.55 (-2.55, 1.45)
Salbutamol	-23.6 (-41.9, -5.3)^$^	-44.6 (-113, 23.8)^#^	1.47 (-0.50, 3.45)
Placebo	-61.0 (-77.9, -44.0)^$^	-66.2(-130, 2.8)^$^	4.20 (2.37, 6.03) ^$^

Data shown as Geometric Mean and 95% confidence interval; AUC_9-17_: Area Under the Curve from 09:00 to 17:00 hours, covering three AMP provocations; changes relative to test-day baseline at 09:00 hours; FEV_1_: forced expiratory volume in 1 second; FEF_25-75_: forced expiratory flow between 25% and 75% of forced vital capacity; comparisons by ANOVA, differences compared to budesonide/formoterol: *p < 0.05, ^#^p < 0.01, ^$^p < 0.001.

**Figure 3:**
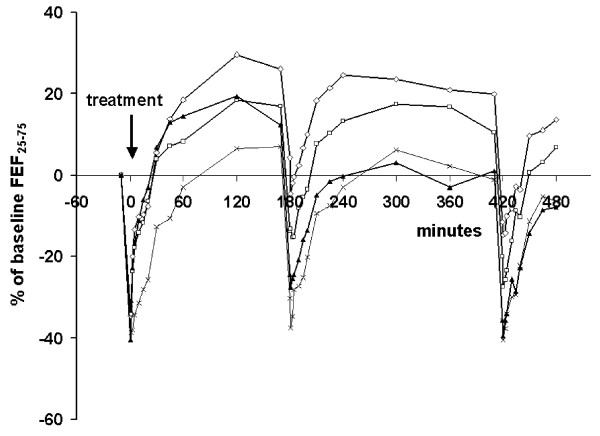
**Mean FEF_25-75 _over the Test Day with three AMP provocations followed by a single dose of budesonide/formoterol 160/4.5 μg (open diamonds), formoterol 4.5 μg (open squares), salbutamol 2 × 100 μg (filled triangles) or placebo (crosses) immediately after the first AMP provocation**.

The time course of FEF_25-75 _over the test day is shown in Figure 3. From 45 minutes onwards, FEF_25-75 _was highest following budesonide/formoterol. The FEF_25-75 _AUC_9-17 _for budesonide/formoterol tended to be greater than that after formoterol (p = 0.070), and differed significantly from that after salbutamol (p = 0.0005) and placebo (p < 0.0001).

**Figure 4:**
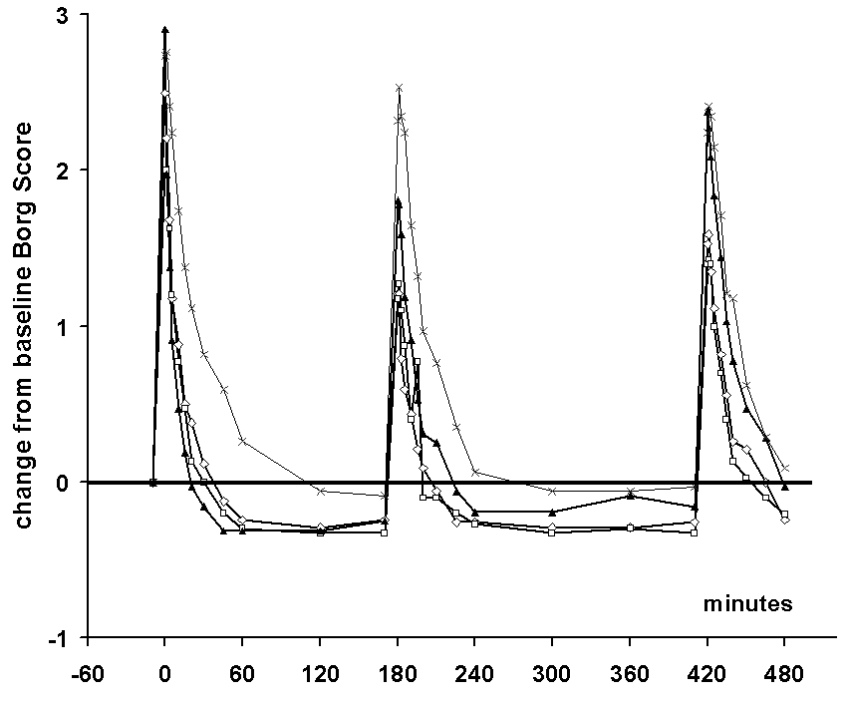
**Mean Borg score over the Test Day with three AMP provocations followed by a single dose of budesonide/formoterol 160/4.5 μg (open diamonds), formoterol 4.5 μg (open squares), salbutamol 2 × 100 μg (filled triangles) or placebo (crosses) immediately after the first AMP provocation**.

The time course of Borg dyspnoea score over the test day is shown in Figure 4. Dyspnoea recovered quickly following all three active treatments. In the third AMP provocation salbutamol had lost its protective effect as assessed with the subjective Borg score whereas both formoterol and budesonide/formoterol had a residual protective effect against AMP-induced dyspnoea. The Borg score AUC_9-17 _after budesonide/formoterol was, however, not significantly different compared with formoterol (p = 0.57) or salbutamol (p = 0.37) but differed significantly from placebo (p = 0.0039).

### Immediate bronchodilating effect

All three active treatments rapidly reversed the AMP-induced bronchoconstriction at 09:00 hours. At 3 minutes after inhalation, the single dose of budesonide/formoterol induced an increase in FEV_1 _of 15.2%, which was statistically significant larger than the 1.7% increase observed after placebo (p < 0.0001), and was comparable to the increase observed with formoterol (13.2%, p = 0.44) but smaller than the 21.5% increase seen after two doses of salbutamol (p = 0.023).

## Discussion

Overall, a single low dose of the combination budesonide/formoterol (160/4.5 μg) had a greater protective effect at 3 and 7 hours after inhalation than a single dose of formoterol 4.5 μg alone, although the difference between these two treatments did not meet the predefined primary endpoint of the maximum % fall in FEV_1 _7 hours after the first AMP challenge. As expected, both treatments with formoterol showed a superior duration of protection relative to the reference treatment salbutamol, which has a shorter half-life of action. These data also support an immediate and lasting additive effect of the inhaled corticosteroid budesonide in protecting against an indirect airway stimulus in asthmatics and provide further rationale for the use of the combination on an as needed basis to prevent further deterioration in case of an asthma exacerbation.

Our study is the first to substantiate the magnitude and duration of the additive protective effect against AMP-induced bronchoconstriction of a low dose of an inhaled corticosteroid on top of a long-acting bronchodilator. Our data add to and are consistent with the previous observation that a single dose of the inhaled corticosteroid fluticasone protects against AMP-induced bronchoconstriction [25,26], and that the effect of a high dose lasts for at least several hours [26]. In a recent study, the budesonide/formoterol combination given immediately after allergen provocation also proved superior to both single components in preventing the late asthmatic reaction as well as the associated increase in bronchial hyperresponsiveness [30].

The study design was intended to mimic an acute asthma exacerbation with multiple AMP provocation tests on single test days. This gave us a unique opportunity to test the contribution of different inhaled drugs, acting via different mechanisms, in this situation. As with every model it has its limitations and does not fully represent a real life asthma attack. Furthermore, because exacerbations can be precipitated by different exposures such as viral infection or allergen exposure, different mechanisms may be involved. In addition, the study was probably slightly underpowered as the sample size estimation was 20, but only 17 patients received treatment and of those only 15 patients had full data available. The additive effect of budesonide on the primary endpoint % fall in FEV_1 _at 7 hours was close to the smallest detectable difference according to the pre-study power calculation (4.4% vs. 4.5% fall) but the standard deviation in the % fall was larger than assumed (10.6% versus 6.8%). On the other hand, for all 3 predefined secondary endpoints with multiple lung function testing the differences were statistically significant.

Ideally, the study would have had an additional study limb in which only budesonide would have been given. This was considered too large a burden for the patients. Additionally, it would not have added to answer our research question on the additive bronchoprotective effect of budesonide on top of the well established effect of formoterol as relief medication.

To explain the observed additive protective effects of budesonide over those of formoterol alone, the potential immediate effects of a corticosteroid on the postulated mechanisms of AMP-induced airway narrowing need to be considered. AMP induces mast cell degranulation and release of mediators leading to airway narrowing due to smooth muscle constriction and mucosal edema as a result of increased mucosal blood flow and increased microvascular permeability [20]. AMP might also act on adenosine receptors in vascular beds and neurosecretory cells to induce mucosal edema directly. Because there is no evidence that a single inhalation of a corticosteroid reduces mast cell number or function, inhibition of mast cell mediator responses is a more likely explanation. In a rat study airway microvascular permeability was shown to be inhibited within several hours after single-dose corticosteroid administration [31]. In addition ICS induce a rapid vasoconstriction by non-genomic effects in asthmatic airways [32,33]. Apparently, these immediate effects of an inhaled corticosteroid on the airway vascular bed provide additional protective benefit over the functional antagonism by formoterol against airway smooth muscle contraction.

Although the latter may be considered a rationale for combining budesonide and formoterol in a single inhaler to be used also for acute asthma symptoms, the clinical relevance might be questioned since the differences between budesonide/formoterol and single formoterol in Borg dyspnoea score over the entire test day were not statistically significant. However, this is most likely because the Borg scores rapidly returned to symptom-free baseline values in between AMP provocations, leaving little room for further improvement. It can be hypothesized that immediate bronchoprotection via multiple mechanisms early during an imminent asthma attack may ameliorate symptoms to such an extent that a full-blown asthma exacerbation is prevented. Support for this can be found in the results of clinical trials that have shown reduced exacerbation rates following use of budesonide/formoterol as maintenance and reliever therapy [10-14] and the efficacy of the combination in the emergency setting [34,35].

In conclusion, the budesonide within the budesonide/formoterol combination inhaler provides additional and sustained protective effects against the external stimulus inhaled AMP in comparison with formoterol alone. In addition, the budesonide/formoterol combination provides immediate bronchodilation when inhaled in a state of bronchoconstriction. This supports the use of this combination for both relief and prevention of asthma symptoms.

## Abbreviations

AMP: adenosine 5'-monophosphate; AUC_0-60_: Area Under the Curve for the 60 minutes after the provocation at 16:00 hours; AUC_9-17_: Area Under the Curve from 09:00 to 17:00 hours; FEF_25-75_: mean forced expiratory flow between 25% and 75% of forced vital capacity; FEV_1_: forced expiratory volume in 1 second; ICS: inhaled corticosteroid; PC_20_: a provocative concentration of AMP causing a 20% fall in FEV_1_; pMDI: pressurised Metered Dose Inhaler.

## Competing interests

RA has received in the last five years honoraria for attendance at advisory boards from AstraZeneca and Novartis totalling €10,000. His department has received the last five years grants from AstraZeneca, totalling to €70,000.

MB is a full-time employee of AstraZeneca, The Netherlands.

HJW has no conflicts of interest.

REJ has received in the last five years travel grants from Bayer, MSD, Boehringer Ingelheim and GSK for attending international congresses.

## Authors' contributions

RA and MB conceived and designed the study. RA, HJW and REJ executed the clinical part of the study. MB supervised the statistical analysis. RA, MB and REJ drafted the manuscript. All authors read and approved the final manuscript prior to submission.
